# Artificial Intelligence can Facilitate Application of Risk Stratification Algorithms to Bladder Cancer Patient Case Scenarios

**DOI:** 10.1177/11795549241296781

**Published:** 2024-11-17

**Authors:** Max S Yudovich, Ahmad N Alzubaidi, Jay D Raman

**Affiliations:** Penn State Health Milton S. Hershey Medical Center, Hershey, PA, USA

**Keywords:** Artificial intelligence, guideline adherence, non-muscle invasive bladder neoplasms, urology, risk factors

## Abstract

**Background:**

Chat Generative Pre-Trained Transformer (ChatGPT) has previously been shown to accurately predict colon cancer screening intervals when provided with clinical data and context in the form of guidelines. The National Comprehensive Cancer Network^®^ (NCCN^®^) guideline on non-muscle invasive bladder cancer (NMIBC) includes criteria for risk stratification into low-, intermediate-, and high-risk groups based on patient and disease characteristics. The aim of this study is to evaluate the ability of ChatGPT to apply the NCCN Guidelines to risk stratify theoretical patient scenarios related to NMIBC.

**Methods:**

Thirty-six hypothetical patient scenarios related to NMIBC were created and submitted to GPT-3.5 and GPT-4 at two separate time points. First, both models were prompted to risk stratify patients without any additional context provided. Custom instructions were then provided as textual context using the written versions of the NMIBC NCCN^®^ Guidelines, followed by repeat risk stratification. Finally, GPT-4 was provided with an image of the NMIBC risk groups table, and the risk stratification was again performed.

**Results:**

GPT-3.5 correctly risk stratified 68% (24.5 of 36) of scenarios without context, slightly increasing to 74% (26.5 of 36) with textual context. Using GPT-4, the model had accuracy of 83% (30 of 36) without context, reaching 100% (36 of 36) with textual context (*P* = .025). GPT-4 with image context maintained similar accuracy to GPT-4 without context, with accuracy 81% (29 of 36). ChatGPT generally performed poorly when stratifying intermediate risk NMIBC (33%-63%). When risk stratification was incorrect, most responses were overestimations of risk.

**Conclusions:**

GPT-4 can accurately risk stratify patients with respect to NMIBC when provided with context containing guidelines. Overestimation of risk is more common than underestimation, and intermediate risk NMIBC is most likely to be incorrectly stratified. With further validation, GPT-4 can become a tool for risk stratification of NMIBC in clinical practice.

## Introduction

Chat Generative Pre-Trained Transformer (ChatGPT) is a popular artificial intelligence (AI) model, which has enjoyed expanded use within numerous medical specialties, including urology. The two versions of ChatGPT most studied include GPT-3.5, a free model, and GPT-4, a powerful subscription-only model. Within the field of urology, there has been a focus on evaluation of ChatGPT with respect to accuracy and quality of responses to general questions regarding various disease processes such as urolithiasis, urologic malignancies, and pediatric urologic conditions. Collectively, these studies have determined that GPT-4 exhibits strong performance when tasked with synthesizing publicly available information.^1-3^ Conversely, additional studies have examined ChatGPT accuracy with respect to answering board-exam questions and have found that GPT-4 performs poorly on specialized examinations.^4,5^

Few urologic studies to date have investigated the performance of ChatGPT with respect to guideline adherence and medical decision-making. Altıntaş and colleagues studied the performance of multiple AI chatbots regarding adherence to European Urologic Association guidelines on urolithiasis with the finding that GPT-4 most consistently aligns with European Association of Urology (EAU) guidelines.^6^ GPT-4 was also found to accurately and adequately answer questions about urinary tract infections according to guidelines.^7^ These studies have involved evaluation and rating of ChatGPT by clinical experts familiar with the guidelines. No studies have examined the ability of ChatGPT to apply urologic guidelines to hypothetical clinical scenarios.

Within gastroenterology, Lim and colleagues studied GPT-4 performance on clinical scenarios when provided with textual context in the form of clinical guidelines as an application for determination of appropriate colon cancer screening intervals.^8^ In this study, ChatGPT was provided with clinical guidelines from multiple sources with the finding that added context improves guideline adherence. For urologic malignancies, the National Comprehensive Cancer Network^®^ (NCCN^®^) provides the NCCN Clinical Practice Guidelines on Oncology (NCCN Guidelines^®^) on risk stratification and management. Non-muscle invasive bladder cancer (NMIBC) is categorized into low-, intermediate-, and high-risk groups based on tumor characteristics.^9^ When provided with generic questions regarding the management of bladder cancer, GPT-4 outperformed GPT-3.5 with generally correct and appropriate recommendations and was found to closely align with the NCCN guidelines.^10^ With respect to diagnosis of bladder cancer, a variety of machine learning and AI tools have been developed, including automated histopathology processing, image interpretation, as well as models to identify genetic aberrations, progression risk, and recurrence risk.^11^ Specific AI image interpretation has been developed for the diagnosis of muscle-invasive bladder cancer; however, they are prone to bias and are not yet thoroughly studied.^12^

The aim of our study is to assess the accuracy of ChatGPT with categorization of hypothetical bladder cancer scenarios using the risk stratification algorithm for NMIBC and to determine whether the presence of context affects response accuracy. This study tests both GPT-3.5 and GPT-4 to determine whether a more powerful large language model is necessary for NMIBC risk stratification.

## Methods

Based on the NCCN risk stratification of NMIBC, a total of 36 hypothetical patient scenarios were manually created with representation of each risk category, tumor histology, and pathologic feature (see Supplement 1).^9^ Each scenario consisted of a description of a hypothetical bladder tumor and included information regarding tumor quantity, size, and histology.

**Figure 1.**
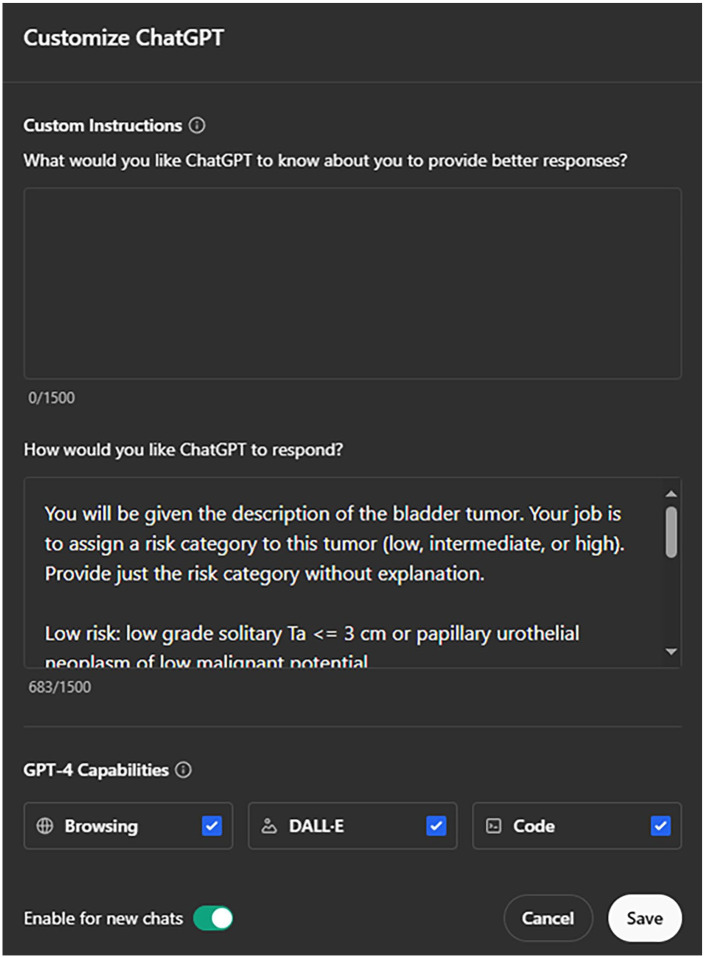
Textual context used for assessment of GPT-3.5 and GPT-4.

**Figure 2.**
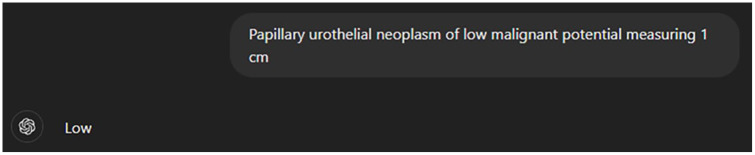
Example prompt with risk stratification.

ChatGPT large language models GPT-3.5 and GPT-4 were used for the analysis. Custom instructions were provided using the “Customize ChatGPT” feature, which consisted of the following: “You will be given the description of the bladder tumor. Your job is to assign a risk category to this tumor (low, intermediate, or high). Provide just the risk category without explanation” (see Figure 1). All tumor scenarios were provided to GPT-3.5 and GPT-4 in separate chat sessions and responses were recorded (see Figure 2). This form of questioning was considered to be without context, as all information regarding risk stratification of bladder tumors was provided through prior training of the language model. All responses were compared with the expected tumor risk as determined by application of the NCCN Guidelines^®^ by study authors.

The custom instructions for ChatGPT were then amended to include written descriptions of the bladder cancer categories with the following information: “Low risk: low grade solitary Ta ⩽ 3 cm or papillary urothelial neoplasm of low malignant potential. Intermediate risk: low grade Ta with recurrence within 1 year, solitary low grade Ta > 3 cm, multifocal low grade Ta, high grade Ta ⩽ 3 cm, low grade T1. High risk: high grade T1, any recurrent high grade Ta, high grade Ta > 3 cm, multifocal high grade Ta, carcinoma *in situ*, BCG failure with high grade, variant histology, lymphovascular invasion, high grade prostatic urethral involvement.” The process of submission of each scenario to GPT-4 was again completed using separate chat sessions using zero-shot learning. This testing scenario was considered to have written instructions for risk stratification context.

Finally, the written descriptions were removed from the custom instructions. During submission of each tumor scenario, an image of the risk stratification table was provided as an uploaded attachment. This testing scenario was classified as having image context.

All data analysis with descriptive statistics was performed in Microsoft Excel. The Fisher exact test was used to compare reproducibility between runs and to assess the benefit of additional context with an alpha level of 0.05. Data were initially collected on October 15, 2023 based on the NCCN guidelines available at the time, and prompts were repeated in August 2024 using the same ChatGPT models without changes to settings or addition of any new outside information.

## Results

**Figure 3.**
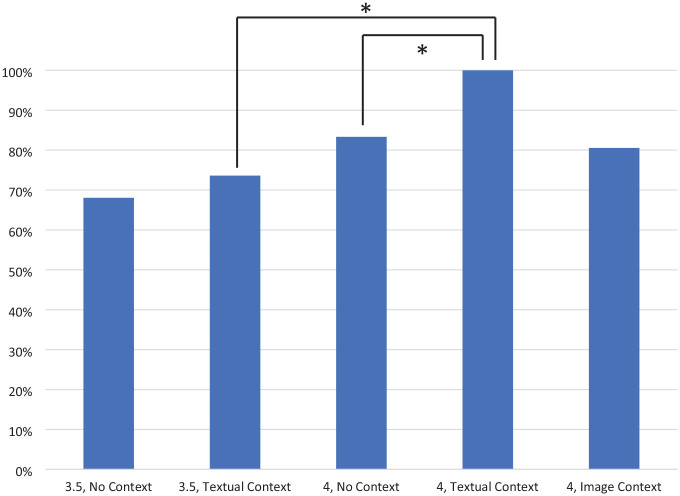
Accuracy of ChatGPT on risk stratification of NMIBC scenarios, by varying degrees of context. Asterisks indicate statistical significance between groups.

Of the 36 scenarios submitted to ChatGPT, 6 were low risk, 15 were intermediate risk, and 15 were high risk. There was no statistically significant difference between the two runs of the scenario data when comparing the performance of similar model versions and context types, indicating temporal reproducibility of the tests. When averaged across both data collections, GPT-3.5 assigned the correct risk stratification for 68% of these scenarios (n = 24.5) without context and 74% (n = 26.5) with textual context (see Figure 3). GPT-4 assigned the appropriate risk stratification for 83% of scenarios (n = 30) without context, which increased to 100% (n = 36) with textual context (*P* = 0.02). The superiority of GPT-4 with context compared with GPT-3.5 with context was statistically significant (*P* = .004). The accuracy of GPT-4 with image context was similar to the accuracy without context at 81% (n = 29). Overall, ChatGPT performed well with risk stratification of low- and high-risk NMIBC (see Table 1). GPT-3.5 without context correctly stratified 75%, 33%, and 100% of low-, intermediate-, and high-risk NMIBC, respectively. GPT-3.5 with textual context, GPT-4 without context, and GPT-4 with image context all answered only 60% to 63% of intermediate-risk NMIBC scenarios correctly.

**Table 1.** Proportion of scenarios in which ChatGPT correctly assigned risk, stratified by context and NMIBC risk.

NMIBC risk	GPT-3.5, no context	GPT-3.5, textual context	GPT-4, no context	GPT-4, textual context	GPT-4, image context
Low	75%	67%	100%	100%	83%
Intermediate	33%	63%	60%	100%	60%
High	100%	87%	100%	100%	100%

Abbreviations: ChatGPT, chat generative pre-trained transformer; NMIBC, non-muscle invasive bladder cancer.

When ChatGPT incorrectly assigned risk, the level of risk was most frequently overestimation, rather than underestimation. Overestimation of risk was defined as assigning intermediate or high risk to a low-risk scenario or assigning high risk to an intermediate-risk scenario. Underestimation was defined as assigning low or intermediate risk to a high-risk scenario or assigning low risk to an intermediate-risk scenario. GPT-3.5 overestimated risk in 28% (n = 10) of scenarios without context and 15% (n = 5.5) of scenarios with textual context (see Table 2). Notably, GPT-3.5 underestimated risk in 11% of scenarios with textual content. GPT-4 underestimated risk in only one scenario. In 14% (n = 5) of scenarios without context and 19% (n = 7) of scenarios with image context, GPT-4 overestimated risk.

**Table 2.** Proportion of scenarios in which ChatGPT incorrectly assigned risk, by varying degrees of context.

	GPT-3.5, no context	GPT-3.5, textual context	GPT-4, no context	GPT-4, textual context	GPT-4, image context
Overestimation	28%	15%	14%	0%	19%
Underestimation	–4%	–11%	–3%	0%	0%

Abbreviation: ChatGPT, chat generative pre-trained transformer.

## Discussion

In this study, we demonstrate that ChatGPT can accurately apply a written version of clinical practice guidelines to hypothetical scenarios. GPT-3.5 underperformed relative to GPT-4 with this difference being more pronounced when textual content was provided. Remarkably, GPT-4 with textual context correctly risk stratified all 36 NMIBC scenarios with close adherence to the NCCN Guidelines. The addition of textual context substantially increased the accuracy of GPT-4; however, visual context in the form of a table interestingly did not. Our observations are similar to Lim and colleagues who previously studied the ability of GPT-4 to follow various society guidelines on assessment of risk factors and determination of appropriate screening intervals for colon cancer screening and surveillance scenarios.^8^ This study found that the ChatGPT model trained with guidelines as context outperformed the model without context. In addition, GPT-4 did not miss any high-risk features in colon cancer scenarios. Similarly, our study demonstrates that GPT-4 with written context did not miss any high-risk bladder cancers.

We also determined that except for GPT-4 with textual context, all models performed poorly during risk stratification of intermediate-risk NMIBC with accuracy rates ranging between 33% and 63%. We suspect that the ease of stratifying the extrema of NMIBC occurs due to the limit quantity of scenarios that qualify as low-risk and high-risk. The category of intermediate-risk NMIBC often exists as a “catch-all” of other tumor qualities, which do not clearly fit the description of low- or high-risk NMIBC. These include high-volume low-grade Ta, low-volume high-grade Ta, low-grade T1, and low-grade Ta recurrence. The multiple conditions placed on tumors within the intermediate-risk group may increase the difficulty of the risk stratification without additional training data.

Hallucination in the realm of AI chatbots refers to incorrect, incomplete, or irrelevant responses to a given prompt.^13^ In our study, hallucination involved assignment of an incorrect risk group to a NMIBC scenario. Although GPT-4 with written context exhibited no hallucination, other forms of context with GPT-4 did have hallucination rates of 17% to 22%. For GPT-3.5, there was a similar number of cases in which NMIBC risk was overestimated (17%) as underestimated (14%); however, with GPT-4, risk was only overestimated. The sole overestimation of risk indicates that if used within a clinical application, GPT-4 would conservatively assign greater risk group to bladder tumors, leading to overtreatment or excess surveillance versus undertreatment with potential for disease progression.

The success of ChatGPT with risk stratification of high-risk NMIBC carries important treatment implications. Patients who have such high-risk tumors with very-high-risk features or BCG-unresponsiveness are often recommended to proceed to radical cystectomy.^9^ Previous studies have identified worsened disease-specific survival for tumors containing variant histology, and variant histology is associated with disease progression after maximal transurethral resection and intravesical BCG.^14,15^ Certain subtypes, including micropapillary, plasmacytoid, small-cell, and sarcomatoid tumors are associated with worse prognostic outcomes.^16^ Other adverse pathologic features such as lymphovascular invasion and prostatic urethral involvement are associated with tumor upstaging and disease recurrence.^17,18^ If AI is used to assist with clinical decision-making, there is risk of underestimating the risk of tumors in which radical cystectomy is indicated, which could lead to worsened outcomes. Therefore, close adherence to clinical principles and updated clinical practice guidelines is required to ensure optimal management.

Our study is a limited demonstration of AI guideline adherence and scenario interpretation. There is only a small quantity of bladder tumors that qualify as low risk, including papillary urothelial neoplasm of low malignant potential and low-volume papillary urothelial carcinoma. On the contrary, a larger quantity of tumors is categorized as intermediate or high risk. Therefore, our study predominantly tested the ability of ChatGPT to recognize intermediate- and high-risk disease. A larger quantity of scenarios could be tested to ensure consistency of the model. In addition, we do not test the ability of ChatGPT to make management decisions. Further studies will be necessary to assess whether ChatGPT can address bladder cancer surveillance intervals or recommend treatments based on risk categorization. It is important to recognize that without clinical validation, AI tools have potential for bias, errors, and gaps in knowledge, which at the present time may result in unsafe patient care. Artificial intelligence in healthcare should be used to augment, rather than replace, clinical expertise.

Clinical guidelines improve standardization of evaluation and management algorithms for health care providers while decreasing variances in patient care. As such, guidelines with high-level evidence have permeated across numerous specialties for benign and malignant diseases. The crux of many guidelines, particularly those in the urologic domain is creation of risk groups based on clinical and pathological features to aid classifying patients. One practical challenge of guidelines implementation is integration into work flow in the clinical environment and modifying physician behavior to encourage guideline adherence.^19^ Specifically, the ability to readily and automatically review patient data to place them into a risk-strata in the setting of a busy clinical environment could afford substantial time savings. Artificial intelligence integration into the electronic health record could assist in clinical management and reduce errors.^20^ In this regard, large language models serve as an invaluable tool to aid the clinician by accurate assignment of risk or patient factors based on easily entered clinical data. Collectively, these resources would greatly augment the clinical care decision-making.

## Conclusions

An emerging application of ChatGPT involves application of clinical guidelines to augment patient care. Our study demonstrates that GPT-4, when provided with context in the form of written society guidelines, can appropriately assign risk to NMIBC case scenarios. GPT-4 outperforms GPT-3.5 at this task, and the addition of written context substantially improved the results. In addition, when risk assessment is incorrect, the risk assigned tended to be higher than expected, reflecting a more conservative treatment approach. These findings suggest that ChatGPT may be used in future to augment and streamline medical decision-making for the treatment of NMIBC.

## Supplemental Material

sj-xlsx-1-onc-10.1177_11795549241296781 – Supplemental material for Artificial Intelligence can Facilitate Application of Risk Stratification Algorithms to Bladder Cancer Patient Case Scenarios Supplemental material, sj-xlsx-1-onc-10.1177_11795549241296781 for Artificial Intelligence can Facilitate Application of Risk Stratification Algorithms to Bladder Cancer Patient Case Scenarios by Max S Yudovich, Ahmad N Alzubaidi and Jay D Raman in Clinical Medicine Insights: Oncology

## References

[1] Cil G Dogan K. The efficacy of artificial intelligence in urology: a detailed analysis of kidney stone-related queries. World J Urol. 2024;42:158. doi:10.1007/s00345-024-04847-z 38483582 PMC10940482

[2] Ozgor F Caglar U Halis A , et al. Urological cancers and ChatGPT: assessing the quality of information and possible risks for patients. Clin Genitourin Cancer. 2024;22:454-457. doi:10.1016/j.clgc.2023.12.017 38246831

[3] Caglar U Yildiz O Meric A , et al. Evaluating the performance of ChatGPT in answering questions related to pediatric urology. J Pediatr Urol. 2024;20:26.e1-26.e5. doi:10.1016/j.jpurol.2023.08.003 37596194

[4] Touma NJ Caterini J Liblk K. Performance of artificial intelligence on a simulated Canadian urology board exam: is CHATGPT ready for primetime? Can Urol Assoc J. 2024;18:329-332. doi:10.5489/cuaj.8800 38896484 PMC11477513

[5] Yudovich MS Makarova E Hague CM Raman JD. Performance of GPT-3.5 and GPT-4 on standardized urology knowledge assessment items in the United States: a descriptive study. J Educ Eval Health Prof. 2024;21:17. doi:10.3352/jeehp.2024.21.17 38977032 PMC11893186

[6] Altıntaş E Ozkent MS Gül M , et al. Comparative analysis of artificial intelligence chatbot recommendations for urolithiasis management: a study of EAU guideline compliance. Fr J Urol. 2024;34:102666. doi:10.1016/j.fjurol.2024.102666 38849035

[7] Cakir H Caglar U Sekkeli S , et al. Evaluating ChatGPT ability to answer urinary tract Infection-Related questions. Infect Dis Now. 2024;54:104884. doi:10.1016/j.idnow.2024.104884 38460761

[8] Lim DYZ Tan YB Koh JTE , et al. ChatGPT on guidelines: providing contextual knowledge to GPT allows it to provide advice on appropriate colonoscopy intervals. J Gastroenterol Hepatol. 2024;39:81-106. doi:10.1111/jgh.16375 37855067

[9] NCCN Clinical Practice Guidelines in Oncology. Bladder cancer. Accessed May 8, 2024. https://www.nccn.org/guidelines/guidelines-detail?category=1&id=1417

[10] Tsai CY Cheng PY Deng JH Jaw FS Yii SC. ChatGPT v4 outperforming v3.5 on cancer treatment recommendations in quality, clinical guideline, and expert opinion concordance. Digit Health. 2024;10:20552076241269538. doi:10.1177/20552076241269538 39148811 PMC11325467

[11] Rossin G Zorzi F Ongaro L , et al. Artificial intelligence in bladder cancer diagnosis: current applications and future perspectives. BioMedInformatics. 2023;3:104-114. doi:10.3390/biomedinformatics3010008

[12] He C Xu H Yuan E , et al. The accuracy and quality of image-based artificial intelligence for muscle-invasive bladder cancer prediction. Insights Imaging. 2024;15:185. doi:10.1186/s13244-024-01780-y 39090234 PMC11294512

[13] Lee P Bubeck S Petro J. Benefits, limits, and risks of GPT-4 as an AI chatbot for medicine. N Engl J Med. 2023;388:1233-1239. doi:10.1056/NEJMsr2214184 36988602

[14] Claps F van de Kamp MW Mayr R , et al. Prognostic impact of variant histologies in urothelial bladder cancer treated with radical cystectomy. BJU Int. 2023;132:170-180. doi:10.1111/bju.15984 36748180

[15] Contieri R Hurle R Paciotti M , et al. Accuracy of the European Association of Urology (EAU) NMIBC 2021 scoring model in predicting progression in a large cohort of HG T1 NMIBC patients treated with BCG. Minerva Urol Nephrol. 2023;75:180-187. doi:10.23736/S2724-6051.22.04953-9 36197700

[16] Claps F Biasatti A Di Gianfrancesco L , et al. The prognostic significance of histological subtypes in patients with muscle-invasive bladder cancer: an overview of the current literature. J Clin Med. 2024;13(15):4349. doi:10.3390/jcm13154349 39124615 PMC11313590

[17] Mari A Kimura S Foerster B , et al. A systematic review and meta-analysis of the impact of lymphovascular invasion in bladder cancer transurethral resection specimens. BJU Int. 2019;123:11-21. doi:10.1111/bju.14417 29807387 PMC7379926

[18] Hernández-Fernández C Herranz-Amo F Moralejo-Gárate M Subirá-Ríos D Caño-Velasco J Barbas-Bernardos G. Infiltrating bladder cancer: prognostic factors, follow-up and treatment of relapses. Actas Urol Esp. 2017;41:352-358. doi:10.1016/j.acuro.2016.07.006 27561847

[19] MacLennan S Duncan E Skolarus TA , et al. Improving guideline adherence in urology. Eur Urol Focus. 2022;8:1545-1552. doi:10.1016/j.euf.2021.10.007 34702647

[20] Harris JE. An AI-enhanced electronic health record could boost primary care productivity. JAMA. 2023;330:801-802. doi:10.1001/jama.2023.14525 37548970

